# A Teledermatology Scale-Up Framework and Roadmap for Sustainable Scaling: Evidence-Based Development

**DOI:** 10.2196/jmir.9940

**Published:** 2018-06-20

**Authors:** Laticha Elizabeth Marolana Walters, Richard Ernest Scott, Maurice Mars

**Affiliations:** ^1^ Department of TeleHealth University of KwaZulu-Natal Durban South Africa; ^2^ Meraka Institute Council for Scientific and Industrial Research Pretoria South Africa; ^3^ Department of Community Health Sciences University of Calgary Calgary, AB Canada; ^4^ NT Consulting – Global e-Health Inc Calgary, AB Canada

**Keywords:** teledermatology, scale-up, Teledermatology Scale-up Framework, TDSF, Teledermatology Scale-up Framework Implementation Roadmap, TDSF-IR, design science research, KwaZulu-Natal, South Africa

## Abstract

**Background:**

The objectives of South Africa’s electronic health (eHealth) strategy recognize the value proposition that telemedicine practices hold for rural and urban referrals, but a lack of accepted and formalized scale-up has impeded realization of benefits. While both synchronous and asynchronous teledermatology exist, these remain localized and not scaled-up. Skin pathology is often the first sign of an HIV/AIDS infection, which remains a major cause of morbidity and mortality in South Africa. It is essential to replace the current inefficient dermatology referral process with a swift, organized, and efficacious one.

**Objective:**

The objective of this study is to present an evidenced-based teledermatology scale-up framework (TDSF) and implementation roadmap (TDSF-IR).

**Methods:**

A qualitative method with a design science research process model was used which consisted of 5 phases: (1) Awareness, which confirmed the need for an evidence-based TDSF and supporting TDSF-IR; (2) Suggestion, where a proposal was delivered on how to develop a TDSF and TDSF-IR; (3) Development, where we identified recommended design requirements and used these to identify and critique existing teledermatology or related scale-up frameworks; (4) Evaluation and validation, where we assessed outputs of the development phase against the design requirements and validated by confirming the veracity of the TDSF and TDSF-IR (validation involved 4 key senior teledermatology stakeholders using a questionnaire with a 5-point Likert scale); and (5) Conclusion, where validation results were used to finalize and communicate the TDSF and TDSF-IR to users.

**Results:**

The study identified 5 TDSF components: eHealth building blocks, eHealth strategic objectives and budget, scale-up continuum periods, scale-up drivers, and scale-up phases. In addition, 36 subcomponents were identified. Each was further characterized and described to enable design of the final evidence-based TDSF. An implementation roadmap (TDSF-IR) was also prepared as a guide for an implementer with step-by-step instructions for application of the TDSF. For the validation study of the TDSF and supporting TDSF-IR, 4 purposively selected key senior teledermatology management stakeholders were asked if they found it useful as a guide to assist the South African public health system with teledermatology scale-up. The mean (SD) of Likert-scale rating was 4.0 (0.53) where 4=Agree and 33 of 36 responses were either agree or strongly agree.

**Conclusions:**

This study developed a TDSF and supporting roadmap (TDSF-IR) that are evidence-based. The proposed approach and described tools could be adapted to assist with ensuring scale-up and sustainability for other eHealth practices in other locations.

## Introduction

The objectives of the eHealth strategy in South Africa recognize the value proposition of telemedicine to address the shortage of specialists in rural hospitals and to improve access to health care [1]. Teledermatology, due to its visual nature, is one of the most common uses of telemedicine [2] and has previously been found to be effective in enhancing access to dermatologists [3]. Similar to other developing countries, South Africa would benefit from sustainable scale-up of its existing teledermatology activities and services, given the high prevalence of significant skin lesions in HIV/AIDS [4,5] and the shortage of dermatologists [6].

Scale-up and sustainability of telemedicine initiatives are long standing issues, and the recent Momentum document on implementing successful telemedicine programs describes these issues [7]. A recent review of teledermatology activities in South Africa has documented both asynchronous and synchronous teledermatology services, some of which have run for over 10 years, but have not yet been scaled up [8]. These circumstances are impeding the realization of the potential benefits of teledermatology services [1,8,9], such as timely triage, diagnosis, and treatment initiation [10] of dermatological manifestations.

A previous study identified the minimum design requirements to inform a conceptual teledermatology scale-up framework (TDSF) using key stakeholder interviews, literature review, program observations, and expert opinion [11]. The minimum requirements were grouped into 4 themes (framework organization, eHealth building blocks, eHealth planning, and eHealth action), which were further separated into 12 categories with 30 requirements [11]. Another study reported that no TDSF existed, and that no eHealth-related scale-up framework met all the design requirements [12].

Despite the recent launch of a teledermatology toolkit [13] and adoption model [14], a gap remains for a conceptual framework supported with an implementation roadmap to assist public health systems with the process of sustainable scaling-up of successful pilots into routine health care. Therefore, there is a need to develop and validate a TDSF, and a roadmap (namely, the TDSF-IR) for its implementation, with measurable scale-up objectives which are aligned to public health system objectives. The framework and roadmap should be objectives realization management-driven, health sector aligned, holistic, and meet the defined TDSF design requirements.

For clarity, some terms need to be defined or described. In the context of this paper, a framework provides a network of interlinked concepts, assumptions, expectations, beliefs, and theories that, together, provide a comprehensive understanding of a phenomenon or phenomena [15], and it lays out key factors, constructs, or variables and relationships among them [16]. Applied to scale-up, and in contrast to spontaneous adoption of innovations, a framework systematically guides the planning and implementing processes, leading to sustained practice [17]. The term scale-up has been defined by the World Health Organization as “deliberate efforts to increase the impact of successfully tested health innovations so as to benefit more people and to foster policy and program development on a lasting basis” [17].

A roadmap (in this study, the TDSF implementation roadmap or TDSF-IR) provides a structured method to guide use of the framework, with step-by-step instructions to ensure a logical flow of inputs and deliverables to achieve the framework’s objectives. Objectives realization management is a means to ensure that the intended top-down national eHealth goals and objectives are met whilst embracing bottom-up provincial strategic goals and objectives. An objectives realization management-driven approach ensures that the intended public eHealth sector goals and objectives are implemented and sustained by using an evidence-based TDSF and roadmap.

This paper is the culmination of studies looking at scale-up of teledermatology in one of the nine Provinces of South Africa and may serve as a model for other developing world implementations. The paper proposes a TDSF and TDSF-IR and describes their final development, validation, and refinement.

## Methods

**Figure 1:**
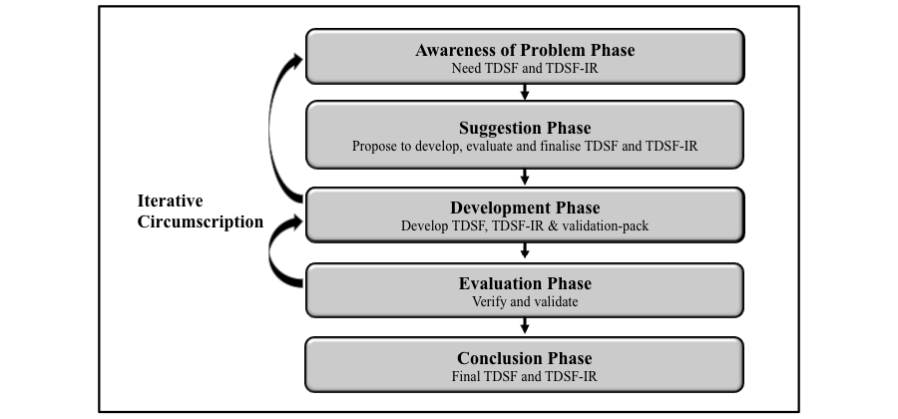
Overview of the design science research process model phases [18] used to meet the main need for an evidence-based teledermatology scale-up framework (TDSF) and implementation roadmap (TDSF-IR).

The design science research process model was adopted [18]. This model consists of 5 phases to solve the research problems and develop knowledge, namely Awareness, Suggestion, Development, Evaluation, and Conclusion (Figure 1). The process is both iterative and compounding, with output from prior phases providing input to succeeding phases, and each phase progressively informing the next, with the conclusion phase completing the process.

The “Awareness” and “Suggestion” phases were complimentary and identified and proposed approaches to resolve the research needs [18]. Evidence for these two phases was gathered through literature review and critique of existing scale-up frameworks. Teledermatology stakeholder interviews, teledermatology program observation, and expert opinion.

During the “Development Phase,” the proposed approaches were used to create specific solutions to meet the needs. This phase consisted of 3 major steps: design and development of a TDSF, design and development of a TDSF-IR, and development of a validation pack.

TDSF Design and Development was based on the previously established design requirements, which were interpreted and organized to define components and subcomponents for the TDSF. Interpretation involved unpacking the themes, categories, and requirement descriptions (including their reasoning and sources) to establish meaning. Thereafter they were organized by determining relationships and dependencies between the components to inform sequencing. In turn, TDSF-IR Design and Development involved creation of a tool to enable implementation of the TDSF. The TDSF-IR was developed by logically describing the organization and processes for implementing components and subcomponents of the TDSF, with estimated durations for completion. Circumscription [19] was used to channel the awareness of new constraints back into the “Awareness of Problem” phase.

The “Evaluation Phase” gave results from piloting the validation-pack with the international expert review panel, before applying the revised pack to key senior teledermatology stakeholders.

The validation-pack comprised a questionnaire and demonstration material to allow the utility of the TDSF and TDSF-IR to be assessed and presented. The questionnaire collected data about respondents’ experience in eHealth or telemedicine or teledermatology (Expert>5 years, Intermediate=2-5 years, Beginner≤2 years), their roles (Advisory, Implementer, User, Manager, Research), 9 validation statements using a 5-point Likert scale (5=Agree strongly, 4=Agree, 3=Unable to assess, 2=Disagree, 1=Disagree strongly), a comments field for every validation statement, and a general comments section at the end (see the Validation questionnaire in Multimedia Appendix 1). The demonstration material consisted of a slide show depicting the TDSF components and their organization during application of the TDSF.

Piloting of the validation-pack used 2 international eHealth experts. The experts were from Canada and Australia and are active in international eHealth research, implementation, and academic fields. The pilot entailed using the validation pack (demonstration material and questionnaire) in one-to-one sessions (1 hour), followed by questions and answers. The experts completed and returned the questionnaires electronically. Based on the experts’ feedback, the validation-pack was revised.

The revised validation-pack was then presented to all 4 key senior teledermatology stakeholders who represented clinical and academic dermatology management and practice, as well as eHealth research. They were selected based on their current teledermatology management roles and their participation in the data collection phase. Except for the eHealth researcher, the key stakeholders represented the current teledermatology management team from the KwaZulu-Natal Department of Health and the Department of Dermatology at the University of KwaZulu-Natal. The management team had also participated in semistructured interviews that identified the TDSF design requirements from an earlier study [11].

Each step of the development phase was also evaluated to ensure the outputs met the associated need. Evaluation entailed verification and validation of the developed artifacts. To verify the design of the TDSF each design requirement was checked to confirm it was mapped to a TDSF component and subcomponent. To verify the TDSF-IR, the step-by-step instructions were reviewed to confirm correct mapping to TDSF action steps and sequencing of deliverables for implementation. Utility of the TDSF and TDSF-IR to assist the KwaZulu-Natal public health management with teledermatology scale-up was determined by using the validation-pack described above.

The final “Conclusion Phase” ensured the artifacts were consolidated into their final form (in accordance with feedback obtained from the evaluation phase), that all contributions were identified, and that the results were clearly communicated.

## Results

### Overview

**Figure 2:**
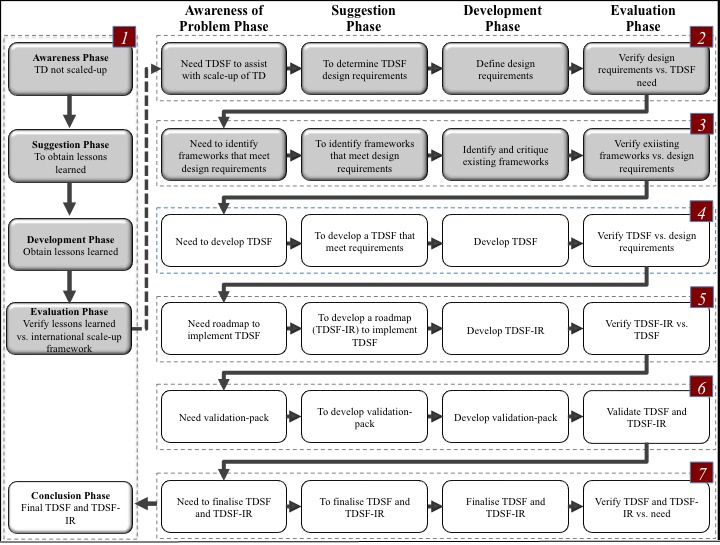
Overview of the complete design science research process model showing phases followed. The shaded areas (steps 1-3) form part of earlier published studies [8,11,12]. TD: Teledermatology; TDSF: Teledermatology Scale-up Framework; TDSF-IR: Teledermatology Scale-up Framework Implementation Roadmap.

The entire process from conception to completion is reflected in Figure 2. Steps numbered 1, 2, and 3 have been previously published [8,11,12] but are included in Figure 2 for completeness. Only results for Steps 4 onwards are described below, that is, those gained through the iterative and reflective process used within the context of the design science research process model and giving rise to specific content.

The results for the developed artifacts are presented in 3 subsections: TDSF, TDSF-IR, and validation of TDSF and TDSF-IR.

### Teledermatology Scale-Up Framework

**Figure 3:**
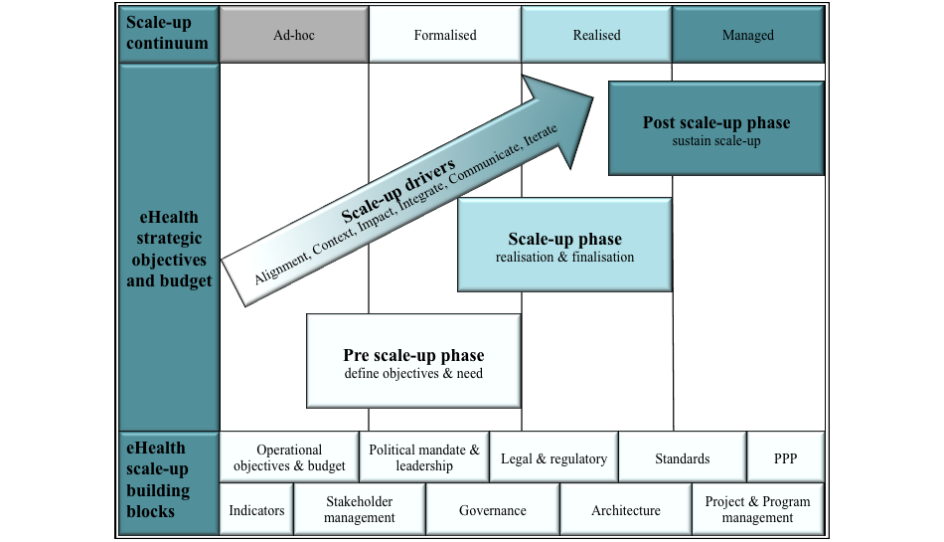
Illustration of how the design requirements fit within the conceptualized Teledermatology Scale-Up Framework (TDSF) and how the scale-up drivers assist with ensuring sustainable scale-up by realizing the teledermatology benefits at each scale-up phase along the continuum, all grounded by critical eHealth building blocks. PPP: Public-Private Partnerships.

The design requirements were interpreted to define 5 components (eHealth scale-up building blocks, eHealth strategic objectives and budget, scale-up continuum, scale-up drivers, and scale-up phases). In addition, 36 subcomponents were identified: 10 within “eHealth scale-up building blocks,” 4 within “Scale-up continuum,” 6 within “Scale-up drivers,” and 13 within “Scale-up activities,” embedded in the 3 “Scale-up phases” (Figure 3).

#### eHealth Scale-Up Building Blocks

Results of performing Step 4 of the Development Phase also included recognition of the need for 10 context specific eHealth scale-up building blocks to form a solid foundation for sustainable scaling. These building blocks are the presence of the following factors listed below:

1. National and provincial government operational objectives and budget
2. Political mandate and leadership
3. Legal and regulatory settings
4. Standards (eg, South Africa’s National Health Normative Standards for Interoperability)
5. Stakeholder management [20]
6. Public private partnerships (PPP)
7. eHealth Performance Indicators
8. eHealth Governance (ie, ICT and Health)
9. Architecture (eg, Health Patient Record System [1])
10. Project and program management capabilities

#### eHealth Strategic Objectives and Budget

The need for official politically— and financially—approved mandates is crucial for scale-up of proven pilot programs.

#### Scale-Up Continuum

The continuum is comprised of 4 periods of evolution extending from “Ad-hoc” actions to “Formalized” actions where governance is influential, to a “Realized” period where specified objectives and benefits are seen, to ultimately the “Managed” period where the entire scale-up process and associated risks are managed (Table 1).

#### Scale-Up Drivers

Ongoing iterative and reflective analysis during Step 4 resulted in identification of 6 scale-up drivers to assist with ensuring sustainable scale-up. These were: intentional alignment, context sensitive, targeted integration, indicator impact, influential communication, and iterative process. These drivers are described in more detail in Table 2. While scale-up activities were related to each specific scale-up phase, the drivers also coincidentally impacted all phases and assisted with moving the process along the scale-up continuum (Figure 3). For example, the pre-scale-up phase activity “Stakeholder management” also had activity associated with each driver (Table 2).

#### Scale-Up Phases

The interpretation process performed during TDSF design and development resulted in the inclusion of 3 scale-up phases (pre-scale-up, scale-up, and post-scale-up). Pre-scale-up activities focused on the definition of scale-up objectives and need activities, scale-up activities focused on scale-up realization and finalization, and post-scale-up activities focused on sustaining scale-up activities. This resulted in organization of the components and subcomponents according to prerequisites to ensure sequential phasing of scale-up activities.

**Table 1:** Description of the scale-up continuum.

Scale-up continuum periods	Description
Ad-hoc	Scale-up planning, implementation, management, and communication is happening on an as-needed basis and is not formally planned, approved and implemented as per TDSF^a^ scale-up phases, activities and steps
Formalized	Scale-up governance processes and structures are planned, approved, communicated, monitored, and controlled as per TDSF pre-scale-up phase, activities and steps
Realized	Scale-up is formalized and functional; agreed health objectives and indicator benefits are realized; and risks are known and actively managed through implementation of plans, communication, monitoring and are controlled as per TDSF scale-up phase, activities, and steps
Managed	Scale-up is formalized, realized, communicated, monitored and controlled as per TDSF post scale-up phase, activities, and steps

^a^TDSF: teledermatology scale-up framework.

**Table 2:** Scale-up drivers and their purpose, areas of application, and (using “Stakeholder management” as an example) relevant activity.

Drivers	Purpose	Areas	“Stakeholder Management” Activity
Intentional alignment	To intentionally support the overall goals and objectives of the health sector	Strategic and Tactical (Operational)	Identify and document the teledermatology stakeholders for public and private sector
Context sensitive	To ensure that the proposed action is appropriate for the health care system and ICT^a^capabilities	Context	Determine the teledermatology stakeholder requirements for key stakeholders (patients, health care system, ICT governance, and architecture)
Targeted integration	To ensure that proposed actions can leverage on existing eHealth^b^interventions	Alignment with existing initiatives	Identify and document existing eHealth stakeholders and assess teledermatology value contribution opportunities to existing relationships
Indicator impact	To ensure that outcomes are measurable, recognised and aligned with health indicators	Sustainability, contribute to bottom line, economic, social and environment	Determine teledermatology’s contribution to the need to increase access to equitable, effective, and efficient health care
Influential communication	To ensure that intent, progress, and contributions are communicated to the right people at the right time	Communicate to all levels of stakeholders at regular intervals	Regularly communicate with key stakeholders such as Department of Health and Health Professions Council the impact and status of teledermatology and request feedback on enabling environment
Iterative process	To ensure that feedback is used to refine and enhance scale-up process along the continuum.	Continuous measure; refine feedback loops to encourage maturity	Assess scale-up status and take recommended action to progress in scale-up continuum

^a^ICT: information and communications technology.

^b^eHealth: electronic health.

### Teledermatology Scale-Up Framework Implementation Roadmap

**Figure 4:**
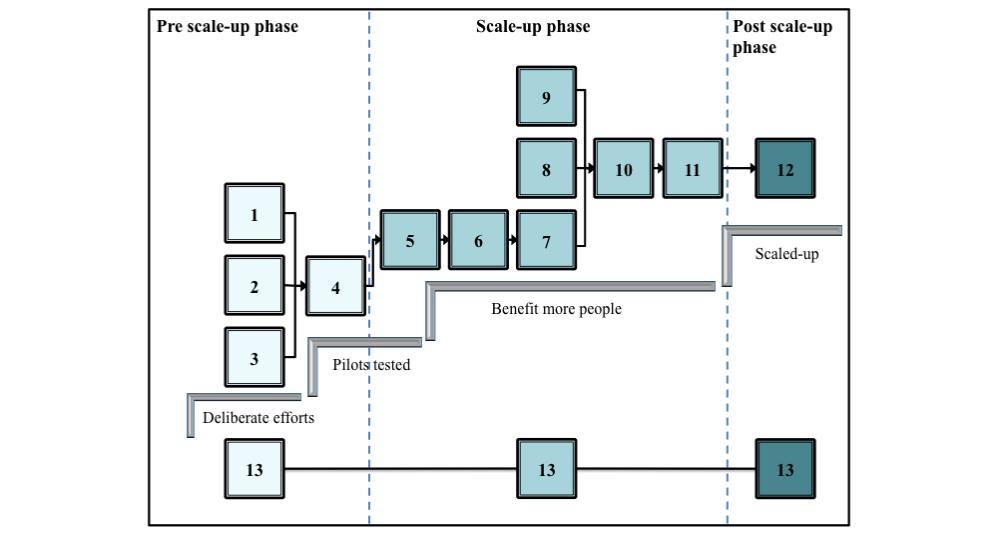
Outline of Teledermatology Scale-Up Framework Implementation Roadmap (TDSF-IR) depicting the relationships and sequencing of TDSF activities through the 3 phases from pre-scale-up to scale-up and post-scale-up. Numbers refer to the 13 TDSF scale-up activities: (1) Define scale-up need, (2) Define scale-up stakeholders, (3) Confirm scale-up compliance, (4) Develop scale-up strategy, (5) Develop detailed scale-up plans, (6) Mobilize scale-up resources, (7) Implement scale-up plans, (8) Manage scale-up benefits, (9) Manage scale-up risks, (10) Confirm scale-up readiness, (11) Finalize scale-up, (12) Manage scale-up sustainability, and (13) Monitor and control scale-up.

The Step 5 Development Phase also resulted in formulation of the content for the TDSF-IR that supports TDSF implementation. The content of the TDSF-IR provides a step-by-step guide to ensure that implementation of the TDSF is executed in a logical sequence (Figure 4).

The sequence was the result of analyzing the dependencies of deliverables (outputs) of one TDSF scale-up activity to provide inputs to the subsequent activity. The 13 TDSF scale-up activities are:

1. Define scale-up need
2. Define scale-up stakeholders
3. Confirm scale-up compliance
4. Develop scale-up strategy
5. Develop detailed scale-up plans
6. Mobilize scale-up resources
7. Implement scale-up plans
8. Manage scale-up benefits
9. Manage scale-up risks
10. Confirm scale-up readiness
11. Finalize scale-up
12. Manage scale-up sustainability
13. Monitor and control scale-up

Within the TDSF-IR activities were mapped to an action plan with 8 fields: activity, inputs (what is needed to start the action), ownership (roles and responsibilities), action (what needs to be done), steps (how to get things done), rules and regulations (policies, standards, procedures and structures), timing (estimated duration in years), and deliverables. The TDSF-IR ensures that the relationship and sequencing of TDSF activities support the implementation process and that deliverables of one activity feed into the next, for example TDSF activities 1, 2, and 3 in Figure 4 provide deliverables (teledermatology scale-up business case, eHealth stakeholder map, and eHealth compliance register) that feed into activity 4.

### Validation of Teledermatology Scale-Up Framework and Teledermatology Scale-Up Framework Implementation Roadmap

The experts recommended the TDSF be more context sensitive to ensure that the needs of South Africa’s public health and key teledermatology stakeholders are clearly addressed. This feedback from the experts was incorporated into the TDSF, and the demonstration process was revised to demonstrate the application of the TDSF to KwaZulu-Natal by using district specific health objectives and indicators. No other changes to the TDSF or TDSF-IR design were required.

The mean (SD) score for the 9 questions in the validation study was 4.0 (0.53) and 33 of 36 responses were either agree or strongly agree. One respondent did not feel that the TDSF could meet the requirements of the KwaZulu-Natal Department of Health, but stated that “I believe that the TDSF can meet the scale-up requirements of KwaZulu-Natal Department of Health” and that:

If properly implemented, TDSF would be able to guide such activities, but I am not sure that the framework on its own can do so. There is a whole eco-system that may impact on various aspect(s) of the framework's realization

The eHealth researcher was unable to assess two questions and was uncertain whether the KwaZulu-Natal Department of Health management would understand the TDSF and whether the Department of Health would be able to leverage on existing eHealth equipment.

Some concerns were raised on the “need to provide more staff dedicated to run the telehealth aspect and training for the Medical Officers and Interns in the respective hospitals” (key stakeholder). It was considered that aspects of staffing are addressed under several components: the pre-scale-up phase (scale-up strategy [resources], eHealth governance [resources]), the scale-up phase (detail planning [change management], mobilization of resources), and the post-scale-up phase (operational plan; building blocks; operational objectives and budget), and no adjustment was made.

## Discussion

### Principal Findings

Using a design science research process model [18] this study addressed the need to use locally identified design requirements to develop an evidenced-based TDSF and supporting TDSF-IR. Each was centered on an objective realization management approach from needs definition through planning, implementation, and finalization, to sustaining scale-up.

The eHealth related scale-up frameworks identified earlier did not meet all the design requirements of the KwaZulu-Natal public health sector [12]. The American Academy of Dermatology [13] launched a toolkit that provides guidance and recommendations for implementers, and Ernst & Young developed a telemedicine adoption model [14,21]. However, neither provided a conceptual framework nor supporting implementation roadmap to assist public health management to ensure sustainable scale-up of successful pilots into routine health care.

While limitations exist (eg, with so few people involved in teledermatology the sample size was restricted and the KwaZulu-Natal Department of Health lacks a telemedicine strategy limiting implementation possibilities), several aspects of the overall design are considered key. The eHealth scale-up building blocks form the foundation of the framework. The building blocks are a prerequisite for sustainable scaling [17] although a risk management approach [22] could be adopted for the absence of any one block. Also, the scale-up phases allow for a phased scaling compared to a big-bang approach where all the activities need to be completed in one implementation cycle. A phased approach to scaling is supported [23] as previously recommended for the TDSF [11]. The scale-up drivers provide the momentum and energy required to push the scaling process. The drivers are designed to guide the implementer through systematic scaling. Finally, the scale-up continuum periods are different from the typical maturity model approach with the intention that scale-up is a process with the goal of integrating teledermatology into routine practice in a sustainable manner. The Health Information System continuum [24] of the WHO compares well with the scale-up continuum although being more applicable for a wider eHealth strategic planning level.

The benefits that a scaled-up teledermatology service holds can now be realized through use of the TDSF and TDSF-IR. Furthermore, the uncommon approach to assist with ensuring sustainable teledermatology scale-up with objectives realization management could potentially assist public health to realize the country’s National Health Insurance goals towards achieving universal health coverage [25].

The proposed evidenced-based conceptual TDSF and supporting TDSF-IR could be considered for future eHealth scale-up framework development and scale-up implementation. The TDSF is based on realizing the objectives defined in strategic plans of South Africa’s National and Provincial eHealth strategic plans (personal communication by Walters LEM, Mars M, Scott RE. An exploration of the use of benefits realisation management in teledermatology related scale-up framework development; 2018).

### Conclusions

A TDSF and TDSF-IR were developed, based on evidence obtained from key stakeholders, program observations, the literature, and the author’s experience, and validated with eHealth management (clinical, academic, research, and general) that rated the TDSF and TDSF-IR as useful to assist the KwaZulu-Natal sector with sustainable teledermatology scaling. These artifacts address a gap in published literature for an evidenced-based teledermatology scale-up framework and supporting implementation roadmap.

The proposed approach and resultant TDSF and TDSF-IR could potentially be adapted to assist with ensuring sustainable scale-up for other eHealth practices in other locations. Future studies could implement the TDSF and TDSF-IR in the real world and use feedback to refine the artifacts.

## Appendix

Multimedia Appendix 1 Validation questionnaire

## Abbreviations

ICT: information and communication technology
TDSF: Teledermatology Scale-Up Framework
TDSF-IR: Teledermatology Scale-Up Framework Implementation Roadmap
