# Supplementary material for: A Teledermatology Scale-Up Framework and Roadmap for Sustainable Scaling: Evidence-Based Development
Source: J Med Internet Res. 2018 Jun 20;20(6):e224. doi: 10.2196/jmir.9940 (PMC6031901; doi:10.2196/jmir.9940)
Supplement: Multimedia Appendix 1 [file jmir_v20i6e224_app1.pdf]

Teledermatology scale-up framework (TDSF) validation questionnaire

Objective of validation session: This questionnaire will be used to measure the usability of the teledermatology scale-up framework (TDSF) in meeting the teledermatology scale-up needs and requirements of KwaZulu-Natal Department of Health (KZN DOH).

About yourself: (please enter the number of years for question 1 and select from the drop-down list for questions 2 and 3)

Questions	Responses
**1. Total number of years in eHealth/ Telehealth/ Telemedicine/ Teledermatology:**	
**2. How would you rate your eHealth/ Telehealth/ Telemedicine/ Teledermatology experience**	Intermediate between 2 and 5 years
**3. How would you describe your eHealth/ Telehealth/ Telemedicine/ Teledermatology role**	Advisory

Instructions:

Please indicate your feedback by double clicking the check box with Agree strongly = 5, Agree = 4, Unable to assess = 3, Disagree = 2, Strongly disagree = 1.

Please provide a reason for selecting **"Unable to assess=3"** by typing your feedback in the column "Comments";

Only select one rating per validation statement.

Ref #	Validation statement	5=Agree strongly	4=Agree	3=Unable to assess	2=Disagree	1=Disagree strongly	Comments
**1.**	I believe that **KZN DOH management** would be able to **understand** the format of **TDSF** (Scale-up continuum, drivers, stages, phases, activities and steps)	☐5	☐4	☐3	☐2	☐1	

Ref #	Validation statement	5=Agree strongly	4=Agree	3=Unable to assess	2=Disagree	1=Disagree strongly	Comments
2.	I believe that **KZN DOH management** would be able to **use** the **TDSF**.	5	4	3	2	1	
3.	I agree that the **TDSF** would enable KZN DOH to **leverage on existing eHealth equipment such as existing videoconferencing equipment in hospitals currently used for tele-education.**	5	4	3	2	1	
4.	I believe that the **TDSF** would be able to **demonstrate teledermatology's value contribution to dermatologist referral system by making the referral process more effective (evidence suggest up to 75% reduction of current referral load) and efficient (proportional saving on ambulances, time away from home and work, scarce dermatologist time).**	5	4	3	2	1	
5.	I am confident that the **TDSF** can **meet the need to assist** KZN DOH with teledermatology scale-up.	5	4	3	2	1	
6.	I believe that the **TDSF** can **meet the defined scale-up requirements of** KZN DOH.	5	4	3	2	1	
7.	I'm confident that **TDSF** provides a **structured process model and implementation guide** for **KZN DOH Management** to **define, plan, implement and operationalise** teledermatology scale-up.	5	4	3	2	1	
8.	I believe that the **TDSF** can be **useful to assist** teledermatology and or eHealth **scale-up** in **developing nations**.	5	4	3	2	1	
9.	I believe that the **TDSF** can be **useful to assist** teledermatology and or eHealth **scale-up** in **developed nations**.	5	4	3	2	1	

Any other comments:

Please note:

Your responses will be recorded and reported on anonymously.
You have the right to withdraw from this research project at any point in time.
